# Supplementary material for: Effects of a level-based immersive virtual reality physical therapy program on static and dynamic balance in Parkinson’s disease: Protocol for a randomized controlled trial
Source: PLoS One. 2026 Mar 16;21(3):e0344457. doi: 10.1371/journal.pone.0344457 (PMC12991210; doi:10.1371/journal.pone.0344457)
Supplement: Attachment — Submitted filename: Response to Reviewers.docx [file pone.0344457.s002.docx]

**Effects of a level-based immersive virtual reality physical therapy program on static and dynamic balance in Parkinson’s disease: Protocol for a randomized controlled trial**

We appreciate your review of our manuscript. We also welcome your comments to improve the quality of the manuscript. We have made the requested changes and revised the manuscript accordingly. Attached is a point-by-point response. We hope you find our responses satisfactory.

**Editor comments:**

The study protocol, while timely and clinically relevant, requires substantial revisions to address several conceptual and methodological issues. Key concerns include the lack of explicit justification for focusing solely on balance and failing to distinguish between static and dynamic balance. The rationale must be strengthened by integrating recent evidence to clarify the added value of IVR. For reproducibility, the intervention description must strictly apply the TIDieR Checklist, detailing exercise progression criteria, the underlying theoretical model, and specific parameters. Critically, the protocol must specify robust safety monitoring procedures for adverse effects like cybersickness, clear discontinuation criteria, and fall procedures. Methodologically, the sample size's effect size must be justified, adherence rules must be explicitly stated, and detail feasibility goals must be included among others. Kindly refer to the reviewer's comments below for further details. The major issue that the are missing details that make it difficult  critically evaluate the intervention at this stage.

**Response:**

Thank you for your comments. A detailed, point-by-point response to all comments from the Editor and Reviewer is provided below, indicating the exact changes made and their location in the revised manuscript.

The protocol is timely and clinically relevant, but several revisions are needed before re-submission. The literature review motivates VR for PD balance yet should more explicitly justify why immersive VR yields incremental benefits and acknowledge cybersickness risks up front.

Design is sensible, but feasibility needs concrete recruitment targets and task thresholds. Methods require cleanup as there are eligibility mismatches (2.5–4 vs 1–4), the authors should harmonize adherence/withdrawal rules, detail safety monitoring (SSQ timing per session, AE/SAE definitions, fall procedures), and ensure intervention reproducibility via fully specified exercise tables, therapist training, and fidelity checks.

The analysis plan should pre-specify linear mixed-effects models for time x group, control multiplicity (e.g., BH-FDR for secondaries), report effect sizes with CIs, and interpret against MCIDs.

**Response:**

In relation to the update of the clinical trials registry, we would like to inform the editor and reviewers that the authors have now obtained the registration code (NCT07274514), following the changes that occurred due to the government shutdown. This registration code has already been included in the manuscript.

In relation to the rationale for IVR and its associated cybersickness risks, we have revised the Introduction and Methods sections to provide more detail on the mechanisms and benefits of IVR for balance rehabilitation in PD, as well as the associated risk of cybersickness. The Introduction now emphasizes that, beyond its technological characteristics, IVR may improve balance by engaging specific sensorimotor and cognitive processes, including rapid visuomotor responses and sensory integration, which are critical for postural control and fall prevention. Evidence from Campo-Prieto et al. (2023) is cited to support this rationale. The Methods section specifies how adverse effects are monitored using the Simulator Sickness Questionnaire (SSQ) after each session, with clear criteria for pausing or discontinuing participation to ensure participant safety.

Concerning the previously identified inconsistencies in eligibility criteria (Hoehn and Yahr stages 2.5–4 vs. 1–4) have been corrected and harmonized throughout the manuscript.

In relation to safety monitoring and falls prevention the Methods section has been expanded to provide a detailed description of safety monitoring procedures. Specifically, we now clarify the timing of the SSQ, which is administered at the end of each intervention session, define how adverse events are monitored and recorded, and describe fall prevention procedures, including continuous one-to-one supervision by an experienced physical therapist and immediate assistance in case of balance loss.

Regarding Intervention reproducibility, to improve reproducibility, the information in the exercises tables has been expanded with specifications, detailing the type of balance as well as the progression criteria and the session structure.

Regarding statistics analysis, thank you for your valuable feedback. We have revised the analysis plan to incorporate your suggestions regarding linear mixed-effects models, reporting effect sizes with confidence intervals, and interpreting results against MCIDs. Specifically, we will use linear mixed-effects models for time × group interactions, report effect sizes with 95% confidence intervals, and interpret findings considering MCIDs for all outcomes. Regarding multiplicity control, we considered BH-FDR; however, given that our secondary outcomes are confirmatory, limited in number, and all comparisons are pre-specified, we will apply Bonferroni correction for post hoc tests. This approach aligns with CONSORT recommendations for trials with a small set of secondary endpoints. We believe these changes strengthen the rigor and transparency of our protocol.

**Reviewers' comments:**

**Reviewer #1: General Comments**

The aim of the study is to evaluate the effects of an immersive virtual reality (IVR) program with balance exercises compared to conventional balance exercises without IVR, with balance as the primary outcome. However, some conceptual and methodological issues need clarification and improvement to strengthen the protocol.

1. Title and Conceptual Focus

The title states that the trial focuses on balance, but the authors do not justify in the abstract or introduction why balance is the central and only outcome highlighted.

**Response:**

Thank you for your comment. We agree that the rationale for emphasizing balance as the primary outcome can be made more explicit. Balance was selected as the central outcome because postural instability is a hallmark feature of Parkinson’s disease (PD) and a major contributor to falls, functional dependence, social isolation, and reduced quality of life. For this reason, balance represents a clinically relevant target for intervention.

While the trial also evaluates gait speed, functional mobility, lower limb strength, and quality of life as secondary outcomes, the title highlights balance to reflect the primary focus of the intervention and its main clinical relevance. To address the reviewer’s concern, we have revised the abstract and introduction to more clearly justify balance as the primary outcome of the study.

2. Missing Rationale and Recent Evidence

The introduction lacks a critical justification of the importance of balance training in Parkinson’s disease and how IVR may modulate specific mechanisms related to balance.

Recent studies should be incorporated to contextualize the protocol within current evidence. In particular:
De Natale et al. (2025), which provides updated insights into the effects of immersive VR in PD and helps identify remaining gaps in the literature. Campo-Prieto et al. (2023), which explored reaction time in IVR as a predictor of falls and adds conceptual depth to the potential mechanisms by which IVR may influence balance and fall risk. Including these papers will help the authors clarify what remains uncertain in the literature and justify the added value of publishing this protocol.

**Response:**

Thank you for your suggestion to incorporate recent literature to strengthen the conceptual framework of the study. Following this recommendation, we have added the study by Campo-Prieto et al. (2023) to the Introduction to provide additional mechanistic context. Although balance was not the primary outcome in that study, its findings linking reaction time in immersive virtual environments with fall risk support the relevance of sensorimotor integration and rapid motor responses, processes that are also fundamental to postural control in PD. With regard to De Natale et al. (2025), while we acknowledge its value as a recent overview of VR-based interventions in PD, this review predominantly focuses on NIVR, and the IVR studies it discusses are already included in our reference list. Therefore, we considered that its inclusion would not substantially add new evidence or conceptual insight specific to IVR-based balance training beyond what is already covered in the current manuscript.

In addition, we have strengthened the Introduction to explicitly highlight the clinical relevance of postural instability in PD, noting that 45–68% of patients experience falls annually. This high incidence of falls, along with the associated loss of functional independence and social isolation, underscores the importance of targeting balance as the primary outcome. IVR may improve balance by enhancing sensorimotor integration, promoting rapid motor responses, and providing real-time multisensory feedback, all of which are fundamental for postural control in PD. Despite existing studies, there remains a gap in the literature regarding protocols that specifically evaluate the effects of IVR on balance as a primary outcome, which this trial aims to address.

3. Intervention Description (TIDieR Checklist Needed)

The rationale for publishing a protocol is to deliver a transparent and reproducible description of the intervention. However, essential aspects are missing.

Authors should apply the TIDieR Checklist, as recommended for clinical trial protocols, to detail:

**Response:**
Thank you for suggesting the use of the TIDieR Checklist. We would like to clarify that the manuscript has been prepared in accordance with the **CONSORT checklist**, which is the most widely used reporting guideline for randomized controlled trials and is explicitly required by *PLOS ONE*. The use of CONSORT ensures transparent, complete, and standardized reporting of the trial design, conduct, and safety procedures.

In addition, we agree that the **TIDieR Checklist** is a valuable framework for reporting clinical trial interventions, and its elements are already addressed in the manuscript as follows:

**Brief name**: Title and Abstract (page 1).

**Why:** Introduction (paragraphs 2–3).

**What (materials):** Methods, section “Intervention” (page 9-10).

**What (procedures):** Methods, section “Intervention” (page 9-10).

**Who:** Section “procedure” (page 8).

**How (mode of delivery):** Methods, section “Intervention delivery” (page 8-10).

**Where:** Added section “Intervention” (page 8).

**When and how much:** Section “Intervention” (page 8-9).

**Tailoring:** Section Intervention (Table 1- Table 2) “Progression and adaptation” (page 10-11).

**Modifications:** Methods (page 10-11).

**How well** (planned fidelity): Methods (page 13).

**How well** (actual fidelity): Methods (page 13).

Whether the protocol targets static balance, dynamic balance, or both. It is unclear whether the authors are referring to static balance, dynamic balance, or both. This distinction should be explicit in the title, abstract, and introduction, given that Parkinson’s disease affects multiple dimensions of balance.

**Response:**

Thank you for your comment. We have revised the title, abstract, and introduction to explicitly state that the protocol targets both static and dynamic balance in people with PD, thereby clarifying the scope of the balance outcomes addressed.

The specific exercises must include: progression criteria and the theoretical model behind progression (e.g., motor learning principles) and exercise parameters according to the FITT-VP model.

Without these details, it is difficult for clinicians and researchers to reproduce or critically evaluate the intervention.

**Response:**

Thank you for your comment. Progression across difficulty levels is grounded in established motor learning principles and is designed to optimize motor adaptation and balance control while minimizing excessive loss of stability. Exercises are task-specific and progressively increase in complexity through reductions in base of support and augmentation of sensory and cognitive demands, consistent with the challenge point framework. The protocol also incorporates variability of practice, augmented feedback provided by the immersive VR environment, and a distributed practice structure. Progression is structured to promote error-based learning, retention, and transfer of balance skills, while the immersive and interactive nature of the intervention enhances attentional focus, motivation, and engagement.

In relation to the **Exercise parameters for the experimental group (FITT-VP):**

- **Frequency:** 2 sessions per week.
- **Intensity:** Highest level achieved without more than one loss of balance in each exercise.
- **Time:** 2–3 minutes per exercise.
- **Type:** Static and dynamic balance exercises, with dual-task demands (cognitive and quiet, proactive and reactive task).
- **Volume:** 6 exercises per session with four difficulty levels.
- **Progression:** Increased task complexity, reduced base of support, increased sensory and cognitive load.

Regarding **Exercises parameters for the control group (FITT_VP):**

- **Frequency:** 2 sessions per week.
- **Intensity:** Highest level achieved without more than one loss of balance in each exercise.
- **Time:** 2–3 minutes per exercise.
- **Type**: Static and dynamic balance exercises, with dual-task demands (cognitive and quiet, proactive and reactive task).
- **Volume:** 8 exercises per session with four difficulty levels.
- **Progression:** Increased task complexity, reduced base of support, increased sensory and cognitive load.

4. Safety Considerations

VR interventions frequently raise concerns regarding dizziness, cybersickness, imbalance, and discomfort. The protocol does not explain:

How the research team will monitor adverse effects?

**Response:**

Thank you for your comment. Adverse effects related to IVR exposure will be systematically monitored at the end of each intervention session using the SSQ, administered by an experienced physical therapist. The SSQ will be used to assess symptoms such as nausea, dizziness, oculomotor discomfort, and disorientation.

SSQ scores and any reported adverse symptoms will be recorded after each session as part of the safety monitoring procedures. This questionnaire will also serve as a criterion for discontinuing participation in the VR physiotherapy program. Participants obtaining a score of 20 will be withdrawn from the study, as this threshold reflects the presence of severe symptoms according to the scale. In addition, if clinically relevant symptoms are identified during a session, the intervention may be paused to ensure participant’s safety. These clarifications have been incorporated into the Methods section.

Criteria for discontinuation or adaptation?

**Response:**

Thank you for your comment regarding criteria for discontinuation or adaptation. At the beginning of each session, participants will undergo a brief 3-minute familiarization phase while seated comfortably, during which the VR headset is adjusted to ensure optimal focus and comfort. During this phase, subjective feedback regarding visual distortion, discomfort, or disorientation will be collected to assess initial adaptation to the immersive environment.

If mild or transient symptoms are reported, the intervention may be adapted by allowing additional rest time, readjusting the VR equipment, or reducing exposure as needed. In addition, the SSQ will be administered at the end of each session. Participants who score 20 on the SSQ will be withdrawn from the study, as this threshold reflects the presence of severe symptoms according to the scale. In addition, the physical therapist will always be attentive to the patient, inviting them to take breaks if necessary.

Together, these procedures constitute predefined and objective criteria for adapting or discontinuing the intervention to ensure participant’s safety.

Whether patients will perform tasks while standing, and how safety will be ensured?

**Response:**

We would like to clarify that fall risk will be actively managed through continuous, direct supervision by an experienced physical therapist throughout all training sessions. The physical therapist will remain in close proximity to the participant at all times, providing immediate physical assistance when necessary and ensuring correct task execution. This approach will be consistent with standard safety procedures in balance training interventions for individuals with PD and has been widely used in previous rehabilitation studies involving populations at high risk of falls. We have clarified this procedure in the Methods section to improve transparency regarding safety and fall prevention during the intervention.

These are critical elements for feasibility and ethical adequacy.

5. Sample Size Justification

The procedure used to calculate sample size is unclear. The authors should justify: Why a specific effect size was chosen? Whether it was based on previous VR studies in PD? A transparent sample size description is essential for protocol validity.

**Response:**
Thank you for your comment. The effect size of d = 0.71 was chosen based on data reported in a previous study evaluating balance performance in individuals with PD using the Mini-BESTest (Conradsson D, Löfgren N, Ståhle A, Hagströmer M, Franzén E. A novel conceptual framework for balance training in Parkinson's disease-study protocol for a randomized controlled trial. BMC Neurol. 2012 Sep 27;12:111. doi: 10.1186/1471-2377-12-111). Specifically, we relied on the standard deviation values for the Mini-BESTest provided in that study, which allowed us to estimate a realistic and empirically grounded effect size for our sample size calculation. This study reported variability metrics that are consistent with the expected performance range in this population and therefore provided an appropriate reference point for our power analysis.

The selected value (d = 0.71) thus represents a conservative, clinically meaningful estimate that aligns both with previous VR findings and with the variability documented in the reference study.
