# Supplementary material for: Effects of a level-based immersive virtual reality physical therapy program on static and dynamic balance in Parkinson’s disease: Protocol for a randomized controlled trial
Source: PLoS One. 2026 Mar 16;21(3):e0344457. doi: 10.1371/journal.pone.0344457 (PMC12991210; doi:10.1371/journal.pone.0344457)
Supplement: Attachment — Submitted filename: Response_to_reviewers_auresp_2.docx [file pone.0344457.s003.docx]

**Effects of a level-based immersive virtual reality physical therapy program on static and dynamic balance in Parkinson’s disease: Protocol for a randomized controlled trial.**

We appreciate your review of our manuscript. We also welcome your comments to improve the quality of the manuscript. We have made the requested changes and revised the manuscript accordingly. Attached is a point-by-point response. We hope you find our responses satisfactory.

**Reviewers' comments:**

**Reviewer #2: General Comments**

The authors present the protocol for a single-blind two arm randomized controlled trial in individuals with Parkinson's disease, evaluating immersive virtual reality (IVR) based balance training versus standard balance training. The manuscript will be strengthened if the authors consider the following points.

1. In the Eligibility criteria section, authors mention that adherence to the intervention protocol below 90% is a reason for withdrawal. They later say that analyses will be based on the intent-to-treat principle, which includes all individuals who have been randomized (so adherence is not a factor). These two statements are inconsistent and should be clarified.

**Response:**

Thank you for your comment. After carefully reviewing your observation, we have decided to remove the criterion regarding withdrawal due to adherence below 90%. We understand that this could lead to confusion, as you correctly pointed out that the analysis will be based on the intention-to-treat principle, which includes all individuals who were randomized, regardless of their level of adherence. Therefore, we have revised the Eligibility Criteria section and removed this reference to ensure consistency with the approach of our analysis.

2. Some clarification is still warranted for determining the difficulty level of the exercises, particularly for those in the IVR group. Authors state that individuals will perform all exercises at the highest possible difficulty level (defined as no more than 1 loss of balance). Part of my confusion may be due to unfamiliarity with testing in PD. Is it true that individuals will start at Beginner level, perform the exercise, and if completed with no more than 1 loss of balance, they will repeat the exercise at the Easy level (and if completed, move on to the Intermediate level, etc.)? Since not all readers will be familiar with PD, some additional explanation may be helpful (for the control group, it seems clear that they will move from one level to the next level, by the "Progression Principle" column in Table 2).

**Response:**

Thank you for this comment. We agree that additional clarification was needed, particularly for readers unfamiliar with exercise progression in Parkinson’s disease. In the IVR group, participants will begin each exercise at the Beginner level and will progress sequentially through increasing difficulty levels if the exercise is completed with no more than one loss of balance. This process will continue until the highest difficulty level that can be safely performed is reached, following the same procedure as for the control group. We have revised the Methods section to explicitly describe this stepwise progression procedure.

3. For the sample size calculation, authors should clarify the correlation between measurements (if they in fact used the repeated measures design). I suspect given that authors state the effect size as "d", they are referring to the difference between groups at a particular time point, but this should be clarified.

**Response:**

Thank you for this comment. We have clarified that the sample size calculation was based on the expected difference between groups at the post-intervention time point (d = 0.71, α = 0.05, 80% power). Although the study includes repeated measurements at three time points, including follow-up, this calculation provides a conservative estimate that is expected to offer adequate power for comparisons at follow-up. The Methods section has been revised accordingly.

4. If authors updated their statistical analysis section to say they are using linear mixed effects models, it isn't clear why they are also mentioning repeated measures ANOVA. Authors will be able to get group differences at the different time points within the linear mixed effects model.

Thank you for this observation. We have removed the reference to repeated‑measures ANOVA from the Statistical Analysis section. The analysis will rely on linear mixed‑effects models, which allow estimation of group‑by‑time differences across all measurement points. The Methods section has been updated accordingly.

5. How will authors report and analyze the adverse events? It seems as though authors only discuss adverse events in the context of IVR? What about adverse events in the control group or adverse events not related to IVR?

**Response:**

Thank you for your comment. Adverse events will be systematically monitored in the IVR group, as this intervention involves a novel technology with known adverse effects. In the control group, the exercises are based on validated programs with no previously reported adverse events, as described in the article by Ernst M, Folkerts AK, Gollan R, Lieker E, Caro-Valenzuela J, et al. Physical exercise for people with Parkinson's disease: a systematic review and network meta-analysis. Cochrane Database Syst Rev. 2023 Jan 5;1(1):CD013856. doi: 10.1002/14651858.CD013856.pub2. Updated in: Cochrane Database Syst Rev. 2024 Apr 08;4:CD013856. doi: 10.1002/14651858.CD013856.pub3. Therefore, systematic monitoring is not planned for the control group. However, any unexpected events reported by participants in the control group will also be documented. For participants in both groups, all adverse events will be recorded in detail, including type, severity, duration, and possible relationship to the intervention.
